# Inhibition of cariogenic biofilms by a zinc-doped phosphate-based glass orthodontic adhesive

**DOI:** 10.1186/s12903-026-09006-x

**Published:** 2026-06-26

**Authors:** Min-Ji Kim, Ji-Young Seo, Utkarsh Mangal, Sung-Hwan Choi

**Affiliations:** 1 Department of Orthodontics, Institute of Craniofacial Deformity, Yonsei University College of Dentistry, 50-1 Yonsei-Ro, Seodaemun-Gu, Seoul, 03722 Republic of Korea; 2 Department of Oral Biology, Yonsei University College of Dentistry, 50-1 Yonsei- Ro, Seodaemun-Gu, Seoul, 03722 Republic of Korea; 3 BK21 FOUR Project, Yonsei University College of Dentistry, Seoul, Republic of Korea; 4 Center for Systems Biology, Massachusetts General Hospital, Boston, MA 02114 USA

**Keywords:** Orthodontic adhesive, Antibiofilm, Zinc-doped phosphate-based glass, White spot lesions

## Abstract

**Objectives:**

To evaluate the anti-biofilm efficacy of an orthodontic adhesive incorporating 9 wt% zinc-doped phosphate-based glass (Zn-PBG) against single-species and multispecies oral biofilms.

**Materials and methods:**

Zn-PBG and phosphate-based glass (PBG) powders were synthesized, and their particle size, density, and elemental composition characterized. The release of calcium (Ca), phosphorus (P), and zinc (Zn) ions from both PBG and Zn-PBG powders was evaluated at various immersion time points. Antibacterial activity against *Streptococcus mutans* (*S. mutans*) was assessed using an insert-based biofilm model comprising three groups: control adhesive without bioactive glass (CTRL), adhesive containing 9 wt% PBG (PG 9), and adhesive containing 9 wt% Zn-PBG (ZnPG 9).

**Results:**

Zn-PBG exhibited sustained Zn ion release, while Ca and P release was lower than that of Zn-free phosphate-based glass. The ZnPG 9 group demonstrated a significant 30.31% reduction in *S. mutans* viability compared to the control group (*P* < 0.001). In the multispecies biofilm model, ZnPG 9 showed significantly reduced biofilm biomass (58.95 ± 14.48 μm³/µm²) compared to the control (89.39 ± 11.63 μm³/µm²) (*P* < 0.05). Additionally, biofilm thickness was significantly reduced (93.71 ± 32.66 μm) compared to the control (172.35 ± 22.64 μm) (*P* < 0.05), along with decreased bacterial density throughout the biofilm depth.

**Conclusions:**

Adding 9 wt% Zn-PBG to orthodontic adhesives effectively prevented both single-species and multispecies biofilm formation by providing ion-mediated antibacterial action. However, these results come from an in vitro model with a short-term (7-day) ion-release assessment, and future research should evaluate long-term effectiveness under clinical conditions.

**Clinical Relevance:**

Zn-PBG-containing orthodontic adhesives may provide a material-based strategy to reduce biofilm accumulation at the bracket–enamel interface, potentially helping to prevent biofilm-associated enamel demineralization during orthodontic treatment without relying on patient compliance.

## Introduction

A major and common side effect of orthodontic therapy is the development of white spot lesions (WSLs), which represent the initial stage of dental caries and can lead to both functional and aesthetic problems [1]. By promoting the accumulation of cariogenic biofilms, the use of fixed orthodontic appliances, such as brackets, bonding agents, and elastomeric rings, rapidly alters the oral environment. The junction where the bracket, adhesive, and enamel converge creates a complex architectural niche that hinders effective biofilm removal through standard oral hygiene practices [2, 3]. These biofilms produce organic acids that lower local pH, leading to enamel demineralization and irreversible damage [4, 5].

To address these challenges, various bioactive fillers have been investigated for their potential to impart therapeutic properties to orthodontic adhesives. Bioactive glasses (BGs) are particularly notable as an excellent alternative to traditional materials due to their ability to provide a sustained and chemically stable release of therapeutic ions [6, 7]. Recent research has focused on enhancing BGs by incorporating antibacterial metal ions such as silver (Ag) and copper (Cu), to inhibit oral pathogens, while maintaining the mechanical integrity of the adhesive [8]. Unlike silicate-based glasses, phosphate-based glasses (PBG) offer a customizable dissolution rate that enables the sustained and controlled release of therapeutic ions [9]. This precise control over ion release is achieved by incorporating network modifiers, which enhance the chemical durability of the glass, while also transforming PBG into a multifunctional platform. PBG can be engineered to deliver diverse biological properties tailored to clinical needs by integrating specific alkaline metal oxide modifiers, such as zinc (Zn), strontium (Sr), or magnesium (Mg) [10, 11].

Among these modifiers, Zn has attracted special attention due to its dual role in both mineralization and antibacterial activity. Zn is a naturally occurring trace element that is primarily found in the outer layers of dental enamel and is essential for its formation and structural modification [12]. Moreover, incorporating zinc into hydroxyapatite, replacing carbonated hydroxyapatite, can promote remineralization of the enamel surface [13]. In addition to their potential for remineralization, Zn ions exhibit strong inhibitory effects on various bacterial processes. The presence of these ions inhibits glycolysis, glucosyltransferase synthesis, polysaccharide synthesis, proton transport across cell membranes, and acid resistance [14]. These effects are partly attributed to the inhibition of F-type ATP synthase (F-ATPase), as Zn ions suppress the activity of key glycolytic enzymes, including glyceraldehyde-3-phosphate dehydrogenase, pyruvate kinase, and phosphoenolpyruvate-dependent enzymes. They also increase proton permeability through bacterial membranes and decrease ATP production in cells that rely on glycolysis [15]. In consequence, zinc-doped phosphate-based glass (Zn-PBG) represents a promising multifunctional additive that offers a synergistic combination of remineralization and biological biofilm resistance. Notably, a previous study found that incorporating 9 wt% Zn-PBG maintained shear bond strength (SBS) comparable to that of conventional adhesives, while ensuring the sustained release of Ca, P, and Zn ions. This specific concentration has been shown to significantly enhance the remineralization of demineralized enamel, as evidenced by increased surface microhardness and the formation of a hydroxyapatite layer [16].

The ability of Zn-PBG-containing orthodontic adhesives to prevent cariogenic biofilm formation, which is the main cause of enamel demineralization, has not yet been studied, despite the fact that their remineralization capability and mechanical compatibility have already been shown. Controlling biofilm at the bracket–enamel interface is crucial to reducing WSLs formation; however, this remains a significant knowledge gap. Therefore, the aim of this study was to evaluate the comprehensive anti-biofilm efficacy of an orthodontic adhesive that incorporated 9 wt% Zn-PBG. The null hypothesis was that the incorporation of 9 wt% Zn-PBG into orthodontic adhesives would demonstrate no inhibitory effects against both single-species and multispecies biofilms.

## Materials and methods

### Preparation and characterization of PBG and Zn-PBG glass powder

Two types of PBG were fabricated for this study: PBG with a composition of 50P₂O₅–30CaO–20Na₂O (mol%), and Zn-PBG with a composition of 38P₂O₅–22.8CaO–15.2Na₂O–24ZnO (mol%). These were synthesized using high-purity precursors, including P_2_O_5_ (99.5%, Sigma-Aldrich, Inc., St. Louis, MO, USA), CaO (98%, Samchun Pure Chemicals Co., Korea), Na_2_O (99.9%, Sigma-Aldrich, USA), and ZnO (99.0%, Sigma-Aldrich, USA). To ensure uniform chemical distribution, the powders were blended for 60 min in a tubular shaker mixer. The mixture was then calcined at 550 °C for 15 min to remove gases, followed by melting in an alumina crucible at 1100 °C for 1 h. Rapid cooling was achieved using a ribbon roller to produce glass cullet, which fragments were initially ground in an alumina mortar. For final particle size reduction, the coarse glass was transferred to a zirconia jar and subjected to dry ball milling in a planetary mono mill (Pulverisette-7, Fritsch, Germany) with zirconia media [16].

The X-ray diffraction (XRD) patterns of PBG and Zn-PBG were recorded using Rigaku Ultima IV diffractometry equipped with monochromatized Cu Kα radiation (λ = 0.154 nm). Data were collected over the 2θ range from 2° to 80° at a scanning rate of 2° min⁻¹ under operating conditions of 40 kV and 30 mA. The particle size distribution of the Zn-PBG powder was determined by laser diffraction particle size analyzer (Mastersizer 2000, Malvern Instruments, UK). The density of the synthesized Zn-PBG was measured in quintuplicate using a helium gas pycnometer (AccuPyc II 1340, Micromeritics Instrument Co., Norcross, GA, USA). This technique is based on the principle of gas displacement, whereby the volume of the solid sample is determined using helium as the displacement medium. The density was subsequently calculated from the measured mass and volume of the specimen. The molar volume was calculated using the experimentally obtained density and the corresponding molecular weight, and the molar volume for each composition was determined according to the equation [17]:

$$V_{m}\:=\:\frac{\sum\:\left({\mathcal{X}}_i\bullet{\mathrm{M}}_i\right)}{\rho\:}$$

where, *ρ* is the glass density, $\:{\mathcal{X}}_{i}$ is the molar fraction of the components *i*, and $\mathrm{M}_{i}$ is the molecular weight of the components *i*. 

### Surface morphology and elemental analysis

The surface morphologies and elemental distributions of both the PBG and Zn-PBG powders were characterized using field-emission scanning electron microscopy (FE-SEM; JEOL-7800 F, JEOL Ltd., Tokyo, Japan) combined with energy-dispersive X-ray spectroscopy (EDS). To ensure surface conductivity and minimize charging effects, the specimens were coated with gold prior to observation. The images were captured at an accelerating voltage of 10 kV and a magnification of 500× to evaluate the shape and surface distribution of the glass particles. In addition, the particle dimensions were directly measured from the SEM images to determine the morphological characteristics of the synthesized powders. To confirm chemical homogeneity, EDS elemental mapping and quantitative analysis were also performed. The atomic percentages of O, Na, P, Ca, and Zn were calculated to verify that the elemental ratios of the synthesized glasses matched their stoichiometric compositions [18]. 

### Ion release analysis of glass powder

The glass powders (PBG and Zn-PBG) were submerged in deionized water at a concentration of 10 mg/mL and incubated at 37 °C to assess their ion release potential. After each specified storage period of (1, 2, 3, and 7) days, the specimens were removed from the solution, and the remaining solution filtered. The collected filtrates were then analyzed using inductively coupled plasma optical emission spectrometry (ICP-OES; Optima 8300, PerkinElmer, Waltham, MA, USA). Following calibration using reference solutions with known concentrations for each target element, this apparatus provided simultaneous multi-element measurement of Ca, P, and Zn ions [19]. 

### Insert-based biofilm model and bacterial viability

#### Sample preparation

To evaluate the antibacterial activity against a single-species biofilm, three groups of specimens were prepared: a control group (CTRL) consisting of pure orthodontic adhesive (Transbond XT, 3 M Unitek, Monrovia, CA, USA), and two experimental groups containing 9 wt% of PBG (PG 9) or Zn-PBG (ZnPG 9) powders incorporated into the same adhesive matrix. All specimens were fabricated using a Teflon mold (10 mm diameter × 2 mm thickness; *n* = 3 per group) [3, 20, 21]. A speed mixer (DAC 150.1 FVZ Speed Mixer, Hauschild, Germany) was used to mix all materials uniformly for 2 min at 3500 rpm [3]. All specimens were made from a single batch to ensure homogeneity and consistency across experimental groups, and the final mixture was covered with opaque aluminum foil to prevent premature light-polymerization. Each specimen was light-cured for 20 s on both sides using an LED light-curing unit. Following polymerization, the surfaces were sequentially polished under running water using #800 and #1200 grit silicon carbide paper to ensure a standardized surface finish for bacterial attachment and subsequent *streptococcus mutans* (*S. mutans*) biofilm formation. 

#### Antibacterial activity against oral single bacterial

To evaluate the antibacterial efficacy against *S. mutans* (KCOM 3065), a biofilm model was established using 24-well plate inserts (SPL insert, SPL Life Sciences, Pocheon, Korea) with a pore size of 0.4 μm. The prepared resin specimens were placed into the inserts, and an *S. mutans* suspension, standardized to an optical density (OD_600_) of 0.1 in brain heart infusion (BHI) broth supplemented with 2% sucrose, was seeded onto the surface of each specimen [22, 23]. The assembly was incubated at 37 °C in a humidified atmosphere containing 5% CO₂. To facilitate biofilm maturation, the culture medium was replenished every 24 h; at 24 and 48 h post inoculation, the spent medium was gently aspirated and replaced with 2 mL of fresh BHI supplemented with 2% sucrose. The biofilms were maintained under these conditions for a total duration of 72 h. Afterward, the specimens were harvested to quantify biofilm. The discs were gently rinsed with 1 mL of phosphate-buffered saline (PBS) to remove planktonic and loosely adhered bacterial cells. To maximize biofilm recovery, each specimen was transferred to 2 mL PBS, vortexed for 30 s, and sonicated at 40 kHz for three cycles of 20 s with 10 s cooling intervals between cycles [24]. The resulting bacterial suspension was serially diluted, and 100 µL of each dilution was spread onto BHI agar plates. After 48 h of microaerophilic incubation, colonies were counted to determine colony-forming units (CFU/mL). 

### Ex vivo biofilm model

#### The formation of multispecies human salivary biofilm

All specimens were categorized into three groups: a CTRL group without BGs, a group containing PG 9, and ZnPG 9. Samples were fabricated using a Teflon mold (10 mm diameter × 2 mm thickness; *n* = 3 per group), and light-cured for 20 s on both sides. Post-curing, surfaces were standardized by sequential polishing under running water with #800 and #1200 grit silicon carbide sandpaper. Biofilm formation was initiated utilizing pooled saliva samples obtained from six healthy adult donors aged 19 years and older (Institutional Review Board approval No. 2 − 2024−0005). Exclusion criteria encompassed pregnancy or lactation, the presence of moderate to severe dental caries, moderate to severe chronic periodontitis, antibiotic usage within the preceding three months, and illiteracy. To reduce inter-individual variability, saliva samples were combined prior to experimental application. Saliva was diluted to 30% in sterile glycerol and stored at − 80 °C. McBain medium containing mucin, peptone, tryptone, and essential vitamins/minerals was prepared and mixed with the saliva in a 50:1 ratio [25, 26]. Each specimen was fully immersed in 1.5 mL of this medium in 24 − well plates, and incubated for 48 h at 37 °C and 5% CO_2_. The medium was refreshed at 8, 16, and 24 h intervals to ensure the development of a robust, well-established biofilm model.

#### Fluorescent staining and confocal analysis

**Fig. 1:**
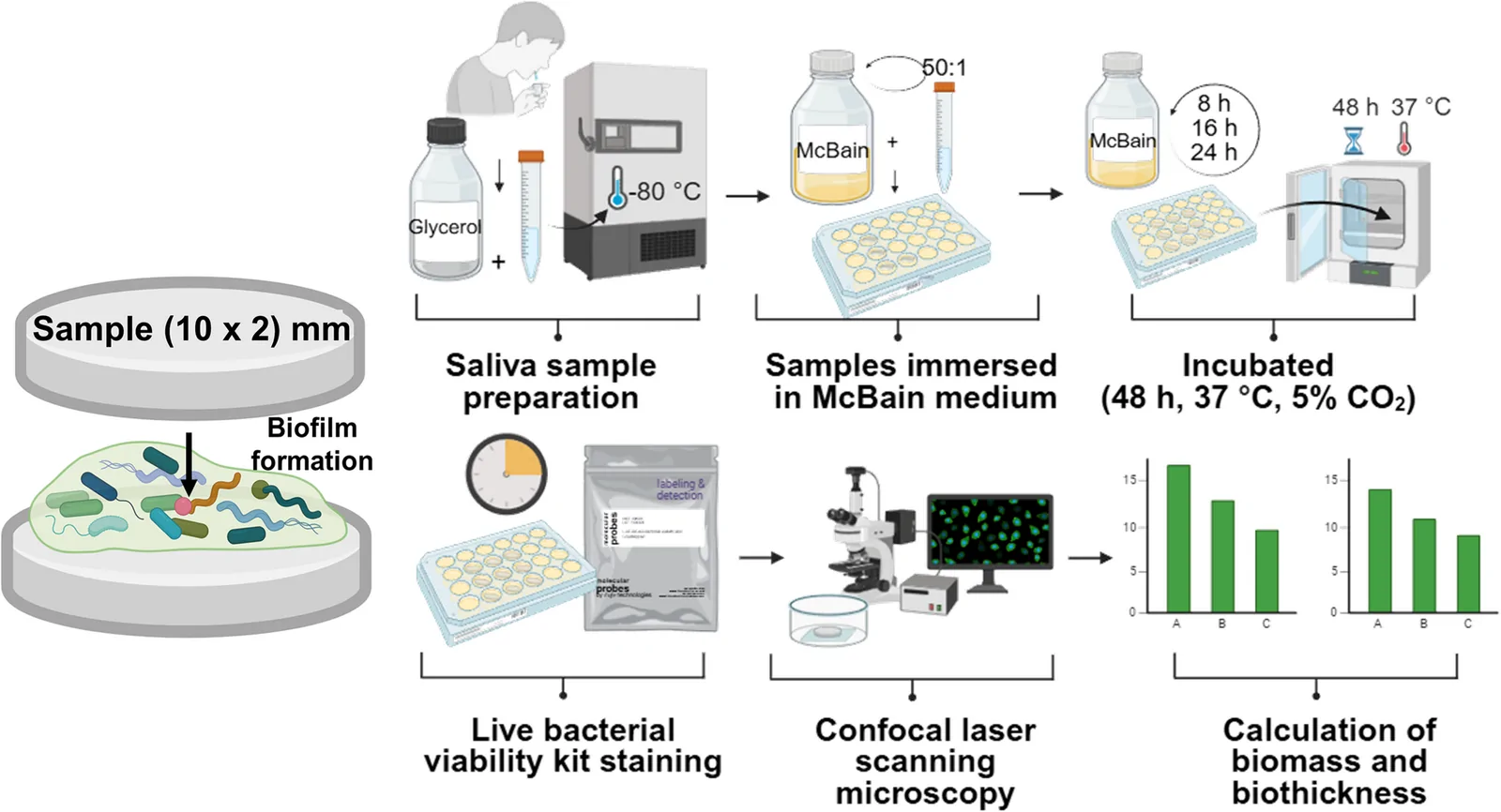
Schematic of the ex vivo protocol using a biofilm model

The experimental protocol for measuring biofilm volume and thickness using a biofilm model is illustrated in Fig. 1. Prior to measurement, specimens were gently rinsed twice with PBS to remove loosely attached microorganisms. Bacteria were then stained using a live bacterial viability kit (Molecular Probes, Eugene, OR, USA) for 15 min, according to the manufacturer’s instructions. Subsequently, EPS staining was employed to analyze the effect on the biofilm extracellular matrix, using ConA (Molecular Probes, Invitrogen Corp., Carlsbad, CA, USA) for staining. The prepared specimens were placed on a cover glass within a confocal dish, and positioned for laser irradiation. The specimens were then examined by confocal laser scanning microscopy (CLSM; LSM980; Carl Zeiss AG, Gena, Germany). The fluorescence wavelengths were set to 488/405 nm for SYTO-9 and EPS fluorescence, respectively. Images were captured using CLSM. For each specimen, three randomly selected fields of view were acquired to ensure spatial representativeness of the biofilm. The COMSTAT plug-in (Technical University of Denmark, Kongens Lyngby, Denmark) in ImageJ (NIH, Bethesda, MD, USA) was used to determine the average biomass. Biothickness was calculated using Zen (Carl Zeiss, Thornwood, NY, USA) software.

### Statistical analysis

All quantitative data were analyzed using OriginPro 2021 (OriginLab Corporation, Northampton, MA, USA). Ion release data obtained by ICP − OES were analyzed using one-way analysis of variance (ANOVA) with Bonferroni-adjusted post hoc multiple comparisons. For antibacterial outcomes, including *S. mutans* CFU counts, as well as confocal-derived biofilm biomass and thickness, group differences were analyzed using one-way ANOVA followed by Tukey’s post hoc test. Statistical significance was set at *P* < 0.05.

## Results

### Characterization of the PBG and Zn-PBG glass powders

**Fig. 2:**
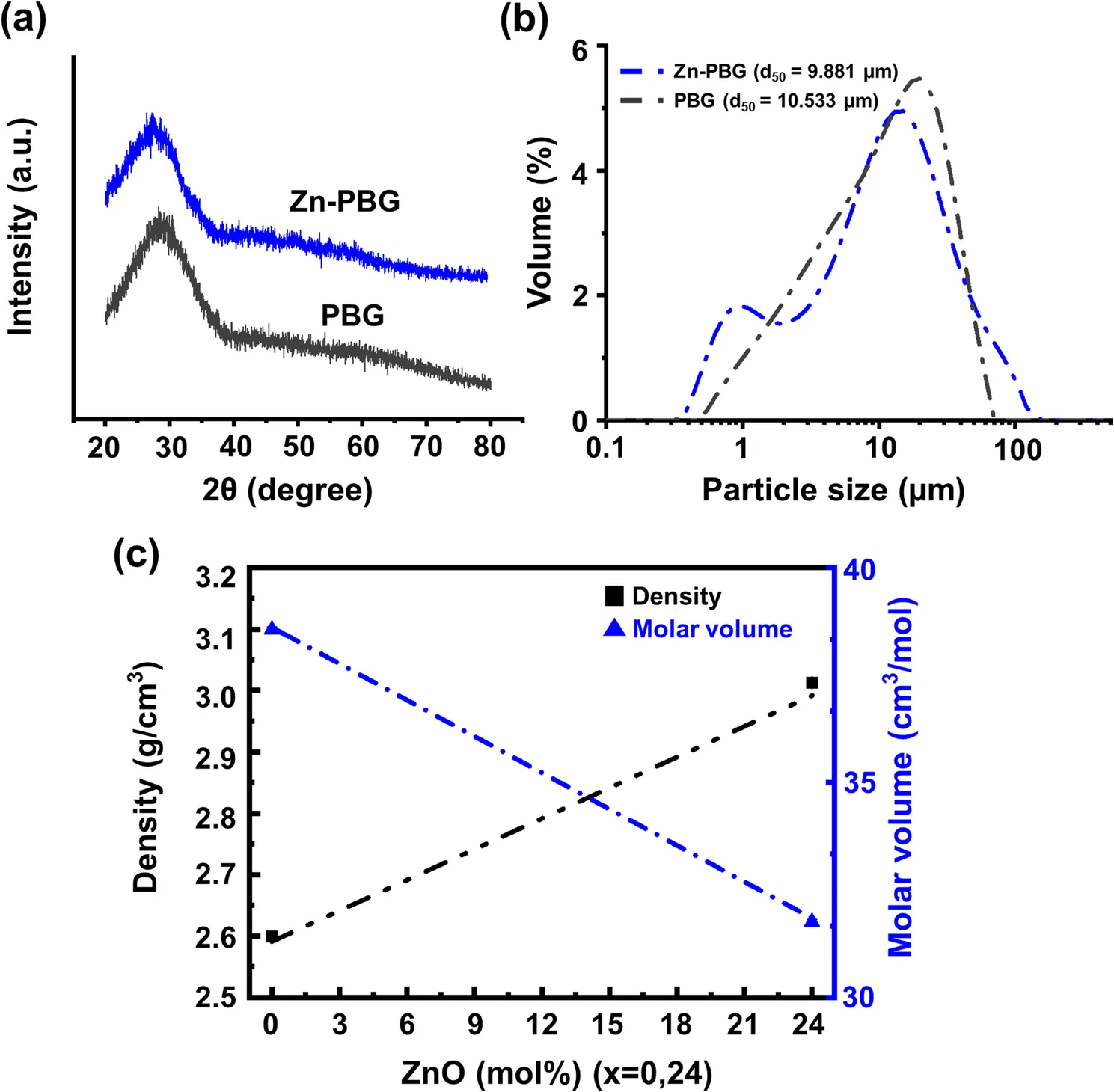
Characterization of the synthesized PBG and Zn-PBG powder. **a **The X-ray diffraction (XRD) patterns, (**b**) particle size distribution, and (**c**) density and molar volume according to the ZnO content

Figure 2a shows the XRD patterns of the produced glass particles. The broad halo peak that both PBG and Zn-PBG exhibit without any sharp crystalline reflections indicates the usual amorphous nature of the glass and the lack of crystalline phases within the glass matrix. Figure 2b presents the particle size distribution analysis of the generated glass powders; the results indicate that the median particle size (D_50_) of the PBG powder was 10.533 μm, while the Zn-PBG powder exhibited a slightly smaller D_50_ of 9.881 μm. Figure 2c presents the density and molar volume of the produced glass powders, categorized by ZnO content. The density and molar volume of the PBG (0 mol% ZnO) were measured as 2.598 g/cm³ and 38.558 cm³/mol, respectively. In contrast, the Zn-PBG (24 mol% ZnO) exhibited a higher density of 3.013 g/cm³ and a lower molar volume of 31.758 cm³/mol compared to the PBG. As the ZnO content increased from the PBG to the Zn-PBG group, a linear increase in density and a corresponding linear decrease in molar volume were observed.

### Surface morphology and elemental analysis

**Fig. 3:**
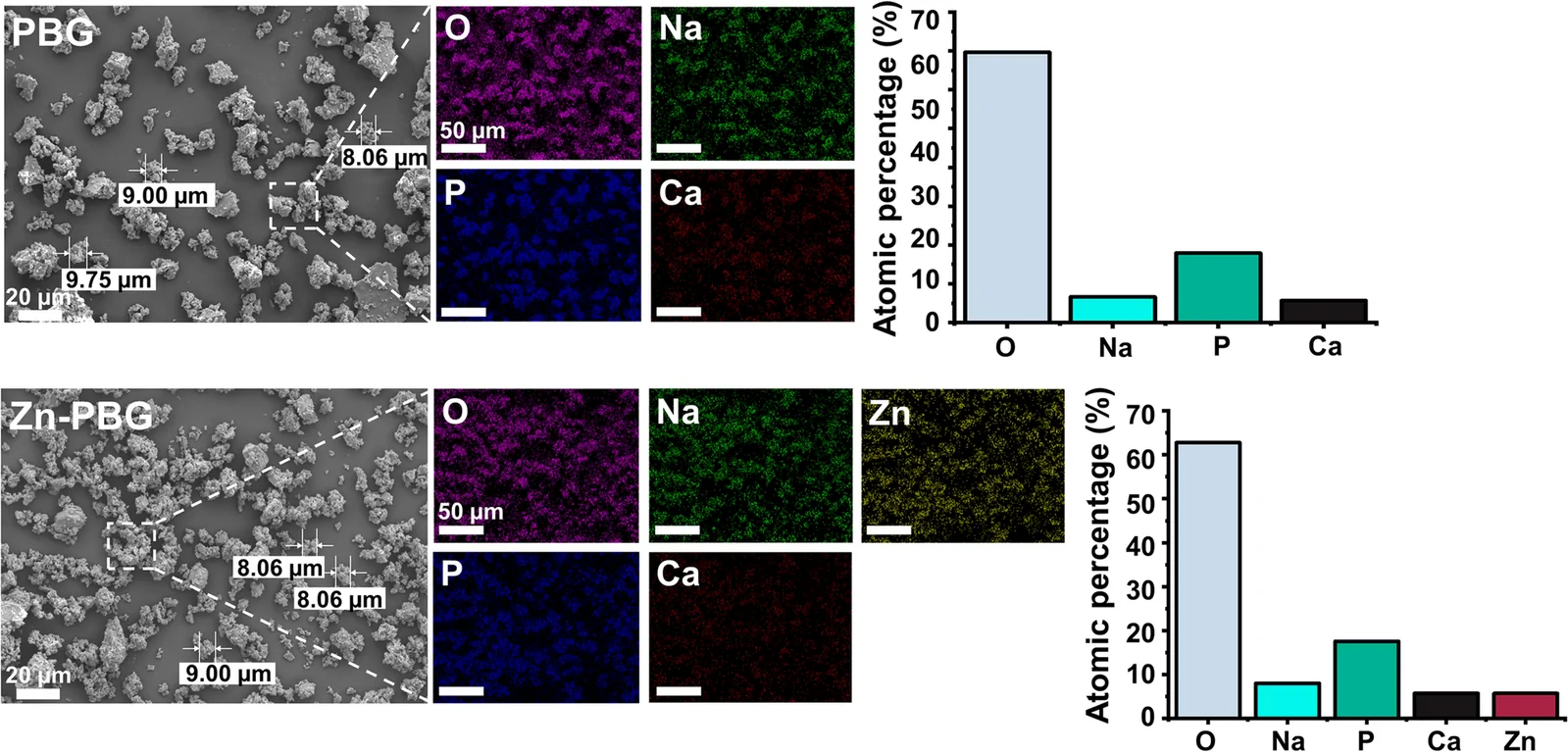
Morphological assessment of scanning electron microscopy (SEM) and Energy-dispersive X-ray spectroscopy (EDS) was used to analyze the elemental composition of PBG and Zn-PBG glass powders, including the distribution of O, Na, P, Ca and Zn. Quantitative EDS results are presented as atomic percentages

Figure 3 presents the surface morphologies and elemental distributions of the glass powders. FE-SEM images revealed that both PBG and Zn-PBG powders consist of irregular, angular particles with various aspect ratios, a morphology typical of pulverized glass produced via melt-quenching. Some aggregates of smaller particles were observed among the larger fragments. Direct measurements from the SEM images showed individual particle dimensions ranging approximately from 8.06 μm to 9.75 μm, consistent with the median particle size results. EDS elemental mapping confirmed the chemical homogeneity of the synthesized glasses. In the PBG group, the base elements O, Na, P, and Ca were uniformly dispersed throughout the particles. Similarly, in the Zn–PBG group, the EDS mapping images clearly demonstrated that Zn was evenly distributed, along with the other constituent elements. The quantitative atomic percentages, illustrated in the accompanying bar charts, show that the elemental ratios closely match the stoichiometric compositions of the glass designs PBG and Zn-PBG.

### Ion release analysis of glass powder

**Fig. 4:**
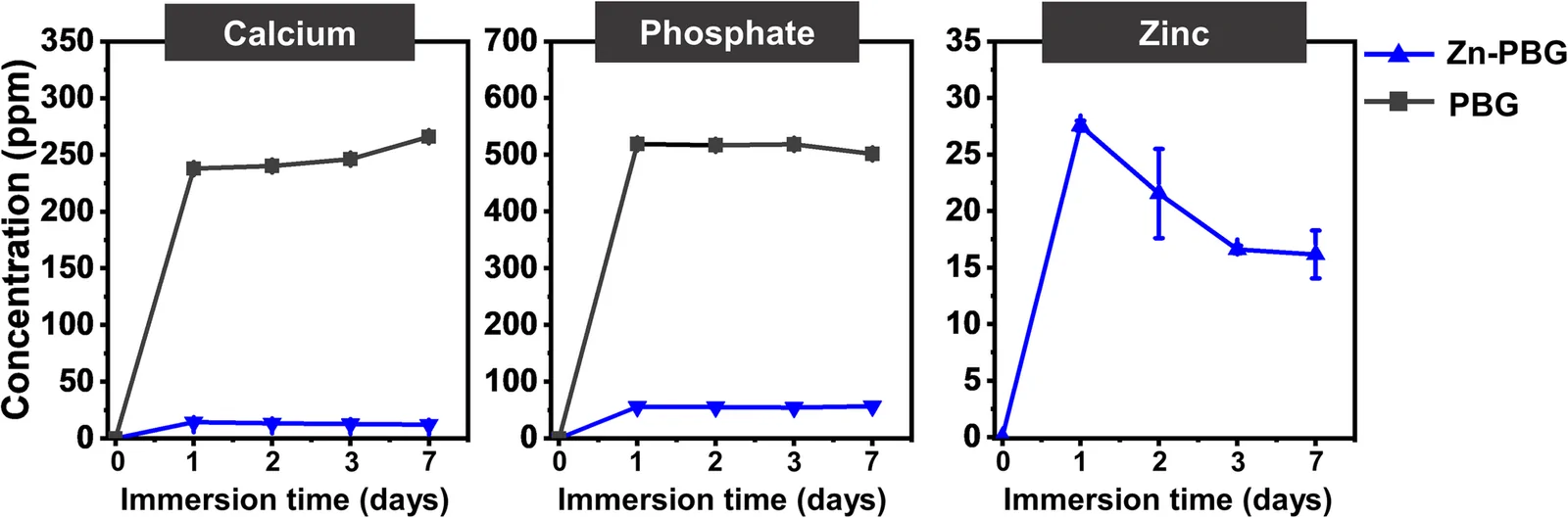
Release profiles of calcium (Ca), phosphorus (P), and zinc (Zn) ions from the PBG and Zn-PBG glass powders as a function of immersion time. Data are presented as the mean value with standard deviation (*n* = 5)

Figure 4 shows the ion release profiles of Ca, P, and Zn ions as a function of immersion time. The Zn ion release from the Zn-PBG group peaked on the first day, then decreased and stabilized over time, while the PBG group showed no Zn ion release, as expected. Ca release from PBG was 237.91 ± 2.80 ppm at 24 h and 265.96 ± 3.30 ppm at 7 days, whereas Zn-PBG released 14.37 ± 0.72 ppm and 12.10 ± 0.59 ppm at the corresponding time points. Similarly, P release from PBG was 519.12 ± 3.70 ppm at 24 h and 501.57 ± 8.57 ppm at 7 days, while Zn-PBG released 55.57 ± 4.17 ppm and 56.53 ± 2.06 ppm at the corresponding time points. The release of Ca and P ions from the PBG group was much greater and faster than that of the Zn-PBG group (*P* < 0.001).

### Insert-based biofilm model and bacterial viability

**Fig. 5:**
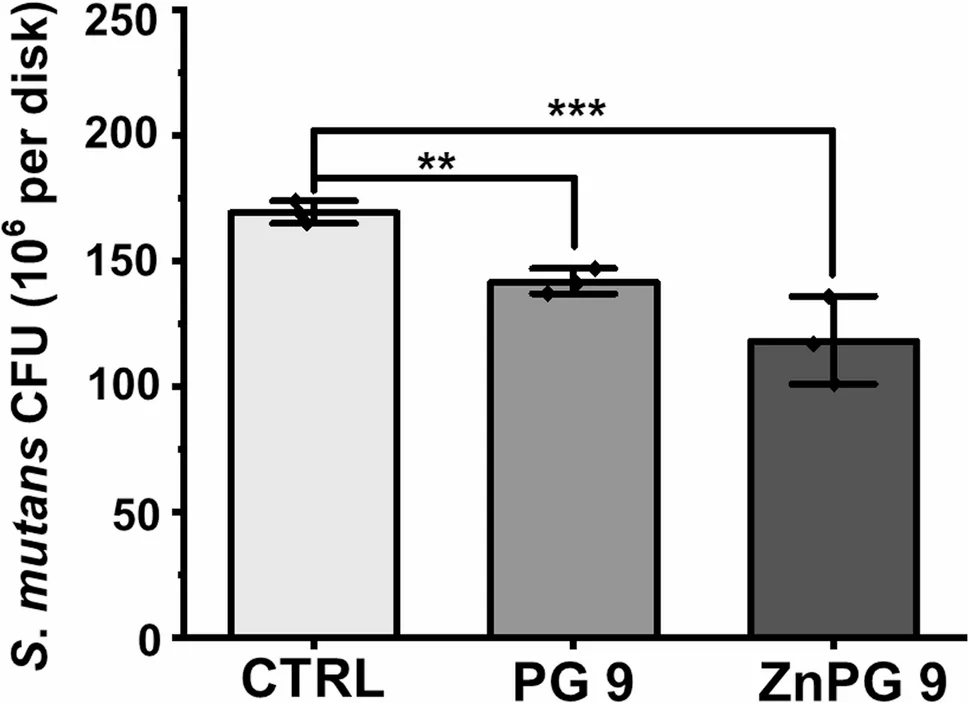
Quantitative analysis of *S. mutans* viability using the insert-based biofilm model. Data are presented as mean values with error bars indicating the standard deviation. Statistical analysis was performed using one-way ANOVA followed by Tukey’s post hoc test. Statistical significance is indicated as (** *P* < 0.01) and (*** *P* < 0.001)

Figure 5 presents quantitative results of the antibacterial efficacy against S. mutans biofilms. The CTRL group showed a mean bacterial viability of (169.33 ± 4.51 × 10⁷ CFU/disk). The PG 9 group (141.67 ± 5.03 × 10⁷ CFU/disk) showed a significant 16.34% reduction compared with the CTRL (*P* < 0.01). Furthermore, the ZnPG 9 group (118.00 ± 17.52 × 10⁷ CFU/disk) demonstrated a significant 30.31% reduction compared to the CTRL (*P* < 0.001).

### Ex vivo multispecies biofilm model

**Fig. 6:**
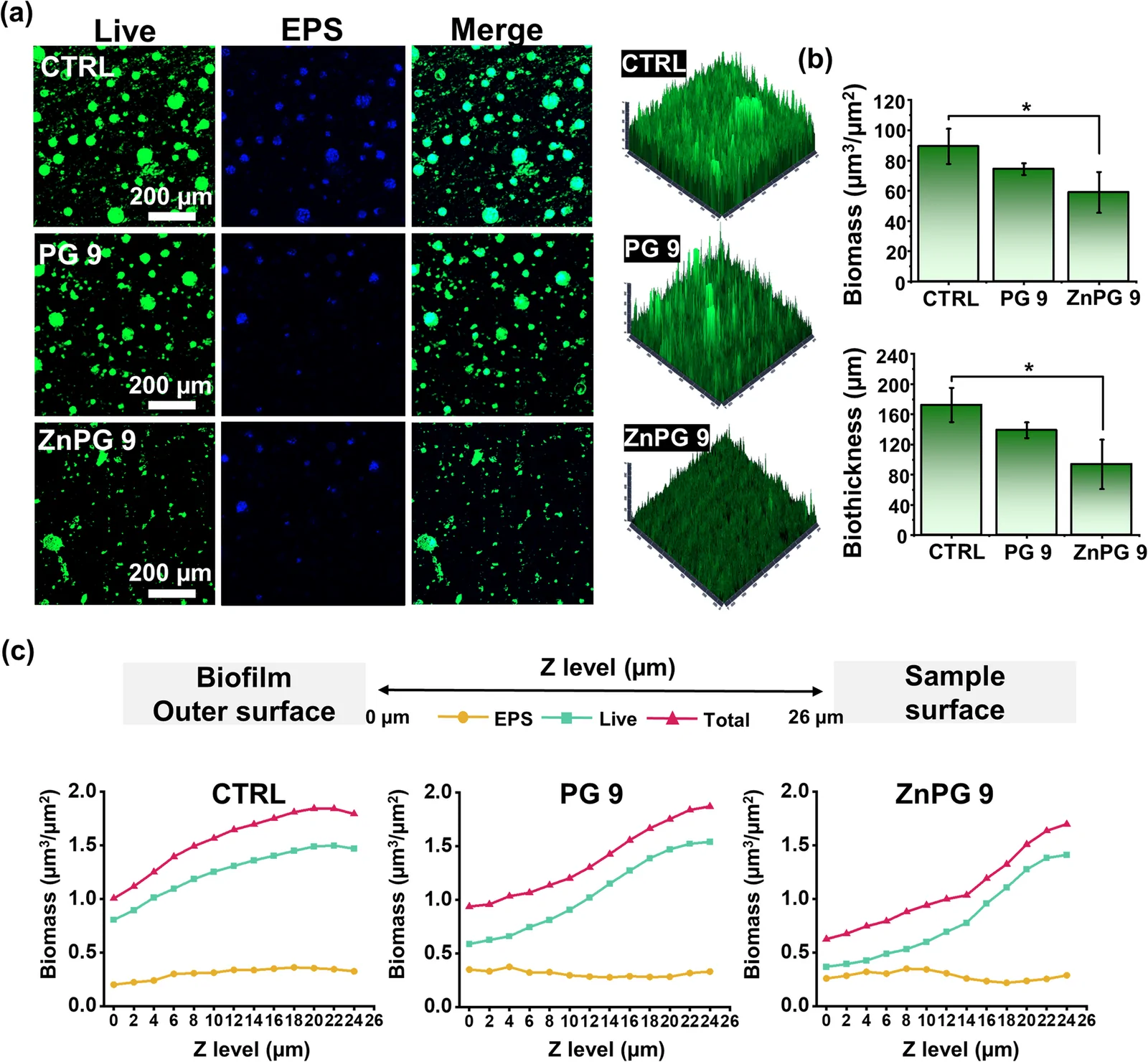
**a **Representative images of live bacteria and EPS of the biofilm attached to each specimen. The scale bar is 200 μm. **b** Quantitative analyses of biomass and thickness are shown. Data are presented as the mean and standard deviation (*n* = 3; * *P* < 0.05). **c** Biomass distribution of bacteria in each layer (Z level) for the CTRL, PG 9, and ZnPG 9 groups

Figure 6a presents the fluorescence live images of the biofilm and the EPS distribution. Compared to the CTRL, the PG 9 group showed a noticeable decrease in green fluorescence and a reduction in EPS distribution. Notably, the ZnPG 9 group exhibited an even more pronounced reduction in both live bacterial cells and EPS compared to the CTRL. In addition, the merged images, which combine the live bacteria and EPS signals, show that the biofilm and its matrix were distributed uniformly within the same space across all groups, while the overall density was markedly lower in the ZnPG 9 group. Figure 6b present the quantitative analyses of the biofilm structural parameters. The ZnPG 9 group (58.95 ± 14.48 µm^3^/ µm^2^) showed a significant reduction (*P* < 0.05) in biomass, compared to the CTRL (89.39 ± 11.63 µm^3^/ µm^2^). Furthermore, the ZnPG 9 group exhibited a 45.63% reduction in average biofilm thickness compared to the CTRL (*P* < 0.05).

To further investigate the density of the architecture, the biomass distribution was analyzed along the Z-levels up to 26 μm from the biofilm’s outer surface to the sample surface (Fig. 6c). Within this basal zone, the ZnPG 9 group exhibited a lower distribution of live bacterial biomass compared to the CTRL and PG 9 groups. In addition, the EPS biomass remained relatively consistent across the 26 μm range for all groups.

## Discussion

The formation of WSLs due to the accumulation of cariogenic biofilms in the complex architectural niches around brackets is a major clinical challenge in orthodontic treatment. To leverage Zn dual functions in mineralization and antibacterial activity, this study focused on incorporating 9 wt% Zn-PBG in orthodontic adhesives. Therefore, the objective of this investigation was to evaluate the overall anti-biofilm efficacy of an orthodontic adhesive containing 9 wt% Zn-PBG.

The two phases of the resin-based dental materials used as orthodontic adhesives are an inorganic filler and an organic resin matrix [27]. Zn-PBG is added to modify the inorganic phase and provide increased bioactivity. Typically, the fillers are used in orthodontic adhesives to make sure the substance can safely debond without causing damage to the enamel surface while also withstanding the applied stresses. In addition, the monomer composition and filler characteristics, such as size, shape, density, and loading concentration, affect the properties of orthodontic adhesive resins [28]. According to previous studies, the BGs content in such materials typically ranges from 1.9 wt% to 9 wt% [29, 30]. Specifically, previous research demonstrated that 9 wt% concentration ensures a shear bond strength (SBS) that satisfies the clinical requirements for reliable bracket bonding. Moreover, the 9 wt% loading was shown to significantly promote enamel remineralization, as evidenced by increased surface microhardness and the successful formation of a hydroxyapatite layer on demineralized enamel surfaces [16]. Thus, this specific loading concentration was selected for the present study to evaluate its comprehensive anti-biofilm efficacy without compromising the fundamental physical properties of the adhesive.

In this study, high-density Zn-PBG was synthesized and its basic properties were evaluated before application to orthodontic resin. First, XRD measurements, which provided information on the crystal phase and structure, confirmed the successful fabrication of the Zn-PBG. The absence of strong reflections in the XRD patterns indicates that Zn-PBG is an amorphous glass [9, 31]. Both powders exhibited a fine and comparable particle size distribution that was appropriate for incorporation into orthodontic adhesives. The minimal difference in D_50_ indicates that Zn incorporation did not substantially influence particle size and these results indicate that Zn addition did not significantly alter the overall particle size distribution. The selection of 24 mol% ZnO for the Zn-PBG filler was based on prior investigations that identified this concentration as the optimal threshold to enhance enamel remineralization while maintaining adequate shear bond strength. Density analysis confirms that incorporating 24 mol% ZnO results in a more compact glass network, as evidenced by the increased density and reduced molar volume. This structural densification is attributed to the high field strength of Zn ions, which reinforce the glass network and ensure the chemical durability necessary for sustained ion release [32–34]. By using this pre-validated composition, the present study focused on evaluating its comprehensive anti-biofilm efficacy. Although in this study, direct mechanical testing was not performed, the observed structural densification of the Zn-PBG filler suggests that it can maintain the physical integrity of the adhesive matrix. This reinforced glass network likely contributes to the chemical stability of the material in the oral environment, ensuring that the therapeutic benefits of ion release are sustained without compromising the bond between the bracket and enamel.

PBG glass dissolving behavior is extremely sensitive to its chemical makeup. Previous research indicates that although the pure P_2_O_5_ is intrinsically unstable, the addition of metal oxides can greatly alter its chemical durability [11]. The release of Ca and P ions from the PBG group was much greater and faster than that of the Zn-PBG group in our ion release results. Ca release from PBG was 237.91 ± 2.80 ppm at 24 h and 265.96 ± 3.30 ppm at 7 days, whereas Zn-PBG released 14.37 ± 0.72 ppm and 12.10 ± 0.59 ppm at the corresponding time points. Similarly, P release from PBG was 519.12 ± 3.70 ppm at 24 h and 501.57 ± 8.57 ppm at 7 days, while Zn-PBG released 55.57 ± 4.17 ppm and 56.53 ± 2.06 ppm at the corresponding time points. This rapid dissolution observed in the PBG group highlights the relative instability of the phosphate network in the absence of the reinforcing effect of Zn. The stabilizing influence of Zn is attributed to its role as an intermediate element that enhances cross-linking within the network [35, 36]. As the ZnO content increases, Zn can act as a network modifier or a network former, creating tetrahedral Zn sites that increase the cross-linking density of the glass [37, 38]. Consequence, the Zn-PBG exhibits greater structural stability than the PBG, and is considered a more suitable glass candidate for therapeutic ion applications. Also, the Zn ion release profile of Zn-PBG shows a peak at 1 day, followed by a gradual decrease in zinc concentration over the subsequent 7 days. This is interpreted as a decrease in ion concentration resulting from the precipitation of Zn(OH)₂, or the recombination of zinc on the glass surface due to changes in pH or saturation of the solution[39]. A cell culture insert system was used to assess the antibacterial activity of the experimental adhesive. This method enabled the continuous exchange of nutrients and released ions through the porous membrane, facilitating the immediate growth of a mature *S. mutans* biofilm on the surface of the resin specimen. In clinical settings, orthodontic adhesives are exposed to a dynamic oral environment that is characterized by fluctuating pH levels, constant salivation, and nutrient replenishment [40, 41]. Since the insert-based culture technique allows frequent medium exchange without disturbing the developing biofilm, it more accurately simulates these conditions. After 72 h of incubation, CFU analysis revealed a significant reduction in viable *S. mutans* biofilm on the Zn-PBG-containing adhesive, compared to the CTRL. The 0.4 μm pore membrane allows continuous ion diffusion from the adhesive into the surrounding medium while preventing bacterial migration through the insert, thereby isolating the biofilm–material interface [42]. Consequently, the observed antibacterial effects can be attributed primarily to ion-mediated biological interactions.

To investigate the bacterial inhibition capability of Zn-PBG, a multispecies biofilm model was employed. An adhesive without glass served as the CTRL, while a PBG-containing group lacking Zn was included to distinguish the specific contribution of Zn ions from the effects of the phosphate-based glass matrix. Compared with both the CTRL and PG 9 groups, the ZnPG 9 group exhibited a reduction in viable bacteria and extracellular polymeric substances (EPS) activity. Bacterial EPS mediate cell-to-cell and cell-to-surface interactions that are essential for biofilm formation, cohesion, and stabilization. A substantial proportion of bacterial species coexist in biofilm communities, which are encased in a self-produced, slimy EPS matrix, and grow attached to surfaces [43]. The EPS matrix provides mechanical stability, offering protection and enhancing cell survivability in changing environments. EPS development is influenced by factors such as pH, temperature, time, and medium composition, with carbon, nitrogen, and divalent ions playing crucial roles. Given that the EPS contains negatively charged functional groups and can bind heavy metal ions, divalent ions like Zn may interact with the EPS, altering its structure and function [44]. In addition, the antibacterial activity of Zn ions, combined with the binding actions of calcium, phosphorus, sodium, and silicon ions, stimulates assimilation reactions [18]. This interaction may fundamentally inhibit EPS formation and, consequently, biofilm development.

Despite the favorable anti-biofilm outcomes observed in this study, several limitations should be acknowledged. Direct mechanical testing and long-term durability assessments, including thermocycling and wear-resistance evaluations, were not performed; therefore, future studies are required to confirm that, during prolonged clinical use, the incorporation of Zn-PBG does not compromise the mechanical performance of orthodontic adhesives. Furthermore, the in vitro nature of the biofilm models used in this study may not fully capture the complexity of the oral environment, where factors such as salivary flow, fluctuating pH, and microbial diversity can influence biofilm behavior and ion release kinetics. Additionally, ion release was assessed from glass powders to characterize the intrinsic dissolution properties of the filler; future studies should evaluate ion release from polymerized adhesive specimens to better reflect clinical conditions. Nevertheless, the clinical relevance of the present findings remains substantial. Building upon a clinically validated Zn-PBG-based formulation, the present study demonstrated that orthodontic adhesives containing 9 wt% Zn-PBG could effectively suppress cariogenic biofilm formation. Furthermore, the sustained release of Ca, P, and Zn ions suggests that this material may provide dual functionality by supporting enamel remineralization while concurrently inhibiting biofilm accumulation. Within these limitations, 9 wt% Zn-PBG orthodontic adhesives represent a clinically relevant, material-based strategy to reduce biofilm-associated demineralization and WSL risk without requiring patient compliance. On this basis, the null hypothesis was rejected.

## Conclusions

This study demonstrates that incorporating 9 wt% Zn-PBG into orthodontic adhesives effectively suppresses biofilm formation by altering bacterial viability and extracellular polymeric substance activity through ion release. The findings indicate that Zn-PBG functions as a therapeutic filler that can modify the local biofilm environment at the bracket–enamel interface, highlighting its potential as a preventive material strategy to manage biofilm-related enamel demineralization during orthodontic treatment.

## Data Availability

The data supporting this study’s findings are available from the corresponding author (Sung-Hwan Choi, email: selfexam@yuhs.ac) upon reasonable request.

## Abbreviations

WSLs: White spot lesions
PBG: Phosphate-based glass
Zn-PBG: Zinc-doped phosphate-based glass
SBS: Shear bond strength
XRD: X-ray diffraction
FE-SEM: Field-emission scanning electron microscopy
EDS: Energy-dispersive X-ray spectroscopy
ICP-OES: Inductively coupled plasma optical emission spectrometry
CTRL: Control
PG 9: Adhesive containing 9 wt% PBG
ZnPG 9: Adhesive containing 9 wt% Zn-PBG
S. mutans: Streptococcus mutans
CFU: Colony-forming units
BHI: Brain heart infusion
PBS: Phosphate-buffered saline
CLSM: Confocal laser scanning microscopy
EPS: Extracellular polymeric substances
ANOVA: Analysis of variance
